# Editorial: Best Practices in Bibliometrics & Bibliometric Services

**DOI:** 10.3389/frma.2021.771999

**Published:** 2021-11-11

**Authors:** Juan Ignacio Gorraiz

**Affiliations:** Department of Bibliometrics and Publication Strategies, University of Vienna, Vienna, Austria

**Keywords:** bibliometrics, scientometrics, bibliometric services, best praclices, informetrics

From my point of view, sciences are very similar to languages. Just as one can speak of dead and living languages, this also applies to the sciences in general and to bibliometrics and scientometrics in particular.

Pritchard already defined bibliometrics as “the application of mathematics and statistical methods to books and other media of communication” in order to “shed light on the processes of written communication and of the nature and course of development of a discipline”.

However, most scientometric journals focus on publishing articles dealing with the introduction of new indicators, the exploration of new methodological techniques, the analysis of new instruments and data sources or the collection and comparison of the results traced from different tools. Contributions of a practical nature showing best practices in different institutions, discussing responsible and sound use of the different metrics, or suggesting new and innovative services for scientists, the administration and science policy makers, are usually rejected despite being of high interest. The reason for the rejection is that they do not contain novel or original research results.

This generates a tendency to favour those scientists who work in their ivory towers and publish an endless number of works without practical use, to the detriment of those ones working from a more practical way, trying to apply correctly indicators and methods, revealing and learning from their deficiencies, and refining and adapting them to suit the needs of the different target groups.

Predominance of theoretical publications makes scientometrics a “dead” discipline, in very clear contradiction with its genuine definition according to Pritchard. A research field is like a language, if it does not find application, it dies. Current research on bibliometrics does not respond to professional needs appropriately. Of course, it should also not only respond to professional needs. Without a solid and innovative theoretical background, we could never build a new discipline and achieve any goal. But, I think that we should also not run the risk of converting bibliometrics in a dead discipline.

To this purpose, it is necessary to bridge the gap between research and professionals conducting bibliometric analyses. We should not forget that science policy and librarian are usually the ones in charge of bibliometric analysis and that, for this reason, their contribution to the discourse is of great importance, as they are best placed to detect problems, benefits and shortcomings in the application of theoretical concepts. But, why is the community still reluctant considering librarians as researchers? Is not “Library and Information sciences” just another discipline more, like religion, politics, economics, or computer sciences?

On the other side, the lack of published examples of practical applications contrasts with the growing number of manifests and recommendations (e.g. San Francisco Declaration on Research Assessment (DORA), Leiden Manifesto, or more recently, the Honk Kong Principles, etc.,) that appear constantly and underlines the need to seek best practices and curb misuse.

However, these initiatives are generally reduced to prevent misuse or give recommendations, instead of providing practical guidance. Therefore, we need concrete examples of responsible use of bibliometrics to be published in order to revive, reinforce and refresh this young discipline.

The purpose of this Research Topic was to gather critical contributions from researchers who are able to share their experiences, initiatives, projects, policies or other insights concerning best practices in bibliometrics. Thus, it provides a short compilation of original applied bibliometric knowledge at the micro-, meso- and macro-level, as well as the description of responsible and innovative bibliometric services. It will also help to refrain from bad practices that are affecting the development of this discipline and contributing to its discredit.

Finally, I would like to thank all the authors for their collaboration and dedication, which was not easy to obtain.

